# Quantification of compensatory motor neuron sprouting at the *Drosophila* larval neuromuscular junction

**DOI:** 10.64898/2026.09.06.749747

**Published:** 2026-09-14

**Authors:** Lizzy Olsen, Aref Zarin

## Abstract

Following nervous system injury, surviving bystander neurons may rewire and form synapses with new postsynaptic targets to restore circuit function. This protocol quantifies compensatory motor neuron sprouting at the *Drosophila* larval neuromuscular junction. We describe larval dissection, immunostaining, Airyscan imaging, image masking, muscle-specific region-of-interest selection, and Fiji-based quantification of Dlg-positive area, bouton number, mean Dlg-positive area per bouton, NMJ arbor length, axonal versus NMJ branch points, ectopic muscle acquisition, and sprouting penetrance.

For complete details on the use and execution of this protocol, please refer to Olsen et al. ^1^

**Graphical abstract:**

**Highlights:**

- Steps for larval dissection, immunostaining, Airyscan imaging, and preprocessing
- Instructions for Fiji-based masking to isolate single-neuron postsynaptic sites
- Guidance on muscle-specific quantification of synaptic area, boutons, and arbor length
- Steps for branch analysis to distinguish axonal sprouting from synaptic remodeling

**eTOC blurb:**

Olsen and Zarin present a workflow for quantifying compensatory sprouting by a single motor neuron at the *Drosophila* larval neuromuscular junction. The protocol integrates Airyscan imaging, Fiji-based image masking, muscle-specific measurements, and branch classification to distinguish new axonal growth from remodeling of established synapses.

## Full Text Availability

The license terms selected by the author(s) for this preprint version do not permit archiving in PMC. The full text is available from the preprint server.

